# Size and number of lymph nodes were risk factors of recurrence in stage II colorectal cancer

**DOI:** 10.1186/s12885-023-10935-x

**Published:** 2023-06-06

**Authors:** Shanyou Tong, Menglei Li, Yichao Bao, Long Zhang, Ping Lu, Tong Tong, Junjie Peng

**Affiliations:** 1 Department of Colorectal Surgery, Fudan University Shanghai Cancer Center, 200032 Shanghai, China; 2 Department of Radiology, Fudan University Shanghai Cancer Center, Fudan University, 200032 Shanghai, China; 3 Cancer Research Institute, Fudan University Shanghai Cancer Center, 200032 Shanghai, China; 4 Department of Oncology, Shanghai Medical College, Fudan University, 200032 Shanghai, China; 5 Department of Colorectal Surgery, Department of Oncology, Shanghai Medical College, Fudan University Shanghai Cancer Center, Fudan University, 270 Dong’an Road, 200032 Shanghai, China; 6 Department of Radiology, Department of Oncology, Shanghai Medical College, Fudan University Shanghai Cancer Center, Fudan University, 270 Dong’an Road, 200032 Shanghai, China

**Keywords:** Stage II colorectal cancer, Lymph node size and number, Recurrence, CT

## Abstract

**Background:**

Size and number of lymph nodes (LNs) were reported to be associated with the prognosis of stage II colorectal cancer (CRC). The purpose of this study was to determine the prognostic role of the size of LNs (SLNs) measured by computer tomography (CT) and the number of retrieved LNs (NLNs) in the relapse-free survival (RFS) and overall survival (OS) among stage II CRC patients.

**Methods:**

Consecutive patients diagnosed with stage II CRC at Fudan University Shanghai Cancer Center (FUSCC) from January 2011 to December 2015 were reviewed, and 351 patients were randomly divided into two cohorts for cross-validation. The optimal cut-off values were obtained using X-tile program. Kaplan-Meier curves and Cox regression analyses were conducted for the two cohorts.

**Results:**

Data from 351 stage II CRC patients were analyzed. The cut-off values for SLNs and NLNs were 5.8 mm and 22, respectively, determined by the X-tile in the training cohort. In the validation cohort, Kaplan-Meier curves demonstrated SLNs (P = 0.0034) and NLNs (P = 0.0451) were positively correlated with RFS but not with OS. The median follow-up time in the training cohort and the validation cohort were 60.8 months and 61.0 months respectively. Univariate and multivariate analysis revealed that both SLNs (training cohort: Hazard Ratio (HR) = 2.361, 95% Confidence interval (CI): 1.044–5.338, P = 0.039; validation cohort: HR = 2.979, 95%CI: 1.435–5.184, P = 0.003) and NLNs (training cohort: HR = 0.335, 95%CI: 0.113–0.994, P = 0.049; validation cohort: HR = 0.375, 95%CI: 0.156-0.900, P = 0.021) were independent prognostic factors for RFS whereas not for OS.

**Conclusion:**

SLNs and NLNs are independent prognostic factors for patients with stage II CRC. Patients with SLNs > 5.8 mm and NLNs ≤ 22 are apt to have higher risk of recurrence.

**Supplementary Information:**

The online version contains supplementary material available at 10.1186/s12885-023-10935-x.

## Background

Globally, colorectal cancer (CRC) is the third most common cancer and ranks second in mortality among all cancers, imposing a heavy burden on patients and society [1]. The characteristics of lymph nodes (LNs) play an important role in the tumor staging system and are strongly correlated with the prognosis of CRC. The features of LNs are increasingly being studied in CRC patients, including size of lymph nodes (SLNs), number of lymph nodes (NLNs), lymph nodes ratio and distribution of metastatic lymph nodes [2–5].

As an early disease, stage II CRC comprises approximately one-quarter of CRC. It’s been reported that the size of retrieved LNs may be a prognostic factor in stage II CRC [2, 6–8].However, the postoperative measurement of retrieved LNs is time consuming, laborious and inaccurate, which may not timely assist physicians with the development of treatment strategy. As the most commonly imaging examination method for CRC, computer tomography (CT) has the advantages of preoperative availability, repeatability, and non-invasiveness. However, most of the limited studies of CT evaluation for LNs are to predict LNs status [9, 10], which cannot be simply applied to stage II CRC. Thus, the prognostic value of SLNs assessed by CT scan for the survival of stage II CRC patients remains unclear.

Although American Joint Committee on Cancer (AJCC) guideline recommends the assessment of at least 12 LNs for stage II CRC [11], the number of LNs assessed in practice range from 7 to 21 [12–17]. Thus, the optimal examined NLNs for stage II CRC is controversial, which is influenced by extent of surgical resection, laboriousness of pathological examination and multiple patient-related factors [18–20].

In this study, we retrospectively investigated patients with stage II CRC at Fudan University Shanghai Cancer Center (FUSCC) to determine the optimal cut-off values and evaluate the prognostic significance of SLNs and NLNs.

## Methods

### Study population

This study retrospectively reviewed 6896 consecutive patients at FUSCC from January 2011 to December 2015. The inclusion criteria were as follows: 18–80 years old; pathologically confirmed primary colorectal adenocarcinoma, mucinous adenocarcinoma, or signet-ring cell carcinoma; stage II according to the 8th edition of the AJCC/UICC TNM staging system; and receiving radical resection of the primary tumor. Exclude the following patients: emergency surgery for acute bowel obstruction, bleeding, or perforation; evidence of distant metastases; neoadjuvant therapy or radiotherapy; history of other malignancies; radiological or follow-up data unavailable. Enrolled 351 patients were randomized into two cohorts at a ratio of 1:1 by a random number table: training cohort and validation cohort, for cross-verification. The training cohort was used to determine the best cut-off point and the validation cohort was used to verify the cut-off value.

This study was approved by the Institutional Review Board of FUSCC and did no harm to patients. All patients provided informed consent. All retrospective data were retrieved from the FUSCC database. Patients were followed up regularly according to Chinese Society of Clinical Oncology (CSCO) guideline. The follow-up data, including recurrence and death, were registered in the Clinical Statistics Center of FUSCC, through the hospital medical records follow-up platform or direct contact with patients via phone or email. Patients alive at the date of analysis were censored at the date of last follow-up.

### CT images acquisition and segmentation

All patients underwent enhanced abdominal or pelvic CT scan according to standard clinical protocol, which were performed on Sensation 64 (Siemens Healthcare, Germany) or Brilliance (Philips Healthcare, Best, The Netherlands) systems. The imaging condition were as follows: 120 kV, 200mA, 5 mm slice thickness, 1.4 or 0.9 pitch, 5.0 mm increment, 512 × 512 matrix and 4.11 cm field of view. All CT enhanced images were collected after 70-75s iodine-based intravenous contrast agent injection. All CT images were retrieved from the picture archiving and communication system (PACS) and exported in digital imaging and communication in medicine (DICOM) format for image segmentation and analysis.

3D regions of interests (ROIs) of LNs were semiautomatically segmented on the venous phase images by an experienced radiologist (M.L.L, with 4-year experience in CRC radiography), using ITK-SNAP software (v3.6.0; www.itksnap.org). The borders of LNs were drawn by excluding adjacent fat, gas, peripheral vascular and normal tissue. To improve the robustness and accuracy of segmentation, an expert radiologist (TT, with 11-year experience in CRC radiography) finally verified and corrected the margins of LNs in the consensus. Then, they measured SLNs independently and any discrepancies were resolved by consensus. To ensure reproducibility, 50 cases were selected and redrawn to test intra-observer consistency using the intraclass correlation coefficient (ICC), with a value of ICC greater than 0.75 indicating good agreement in feature extraction. To ensure the accuracy of LNs masking, the LNs mask was evaluated by the chief radiologist, T.T.), according to the same guideline used to define the boundary of LNs. The parameters measured by the observers showed good agreement, with a mean ICC of 0.88.

**Fig. 1:**
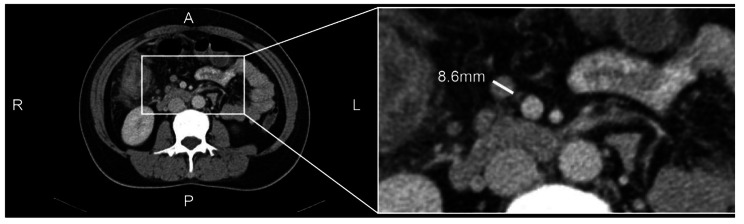
An example case of size measurement of lymph nodes. The largest short diameter was measured on an axial slice that contains the largest diameter of the lymph node

The short-axis diameter of a LN was measured as it had been demonstrated that this was constant despite orientation. The short-axis diameter was measured perpendicular to the longest diameter of the LN, on the axial slice that demonstrated largest cross-sectional diameter of the LN. The largest short-axis diameter of all LNs was recorded and referred to as the SLNs for each patient (Fig. 1). The two radiologists were blinded to any clinicopathological information including treatment details.

### Evaluation of number of lymph nodes

All patients were operated by two or three experienced colorectal surgeons and underwent open or laparoscopic surgery. All pericolic nodes, intermediate nodes, and main nodes were dissected, according to a standardized protocol. Specifically, LNs were identified by direct inspection and manual palpation after closely slicing the mesocolon and mesorectum tissues. No additional fat clearance or methylene blue injection techniques were performed. LNs were fixed in formalin and were stained with hematoxylin and eosin. All specimens considered candidate LNs were examined by experienced pathologists. NLNs were obtained by review patients’ pathology reports.

### Statistical analysis

Statistical evaluation was performed using IBM SPSS version 25(SPSS Inc., Chicago, IL, USA) and GraphPad Prism version 7 (La Jolla, CA, USA). X-tile program (Version 3.6.1, Yale University, New Haven, CT, USA) [21] was used to generate the optimal cut-off values for SLNs and NLNs in the training cohort. The data including SLNs (mm), NLNs, OS events (designated as 0 for no death event, 1 for death event), OS months, RFS events (designated as 0 for no relapse event, 1 for relapse event) and RFS months were inputted to X-tile program and X-tile program identified the cutoff with the minimum P-values from log-rank Chi-square statistics for the SLNs and NLNs in terms of survival. Categorical variables were compared with the two-sided Pearson Chi square test, or Fisher’s exact test as appropriate. The SLNs was treated as a continuous variable and compared with the t test or the Wilcoxon rank test when appropriate. Survival analysis was performed using the Kaplan-Meier method, the log-rank test and Cox regression model. All tests were two sided and P values < 0.05 were considered statistically significant.

## Results

### Baseline characteristics

**Fig. 2:**
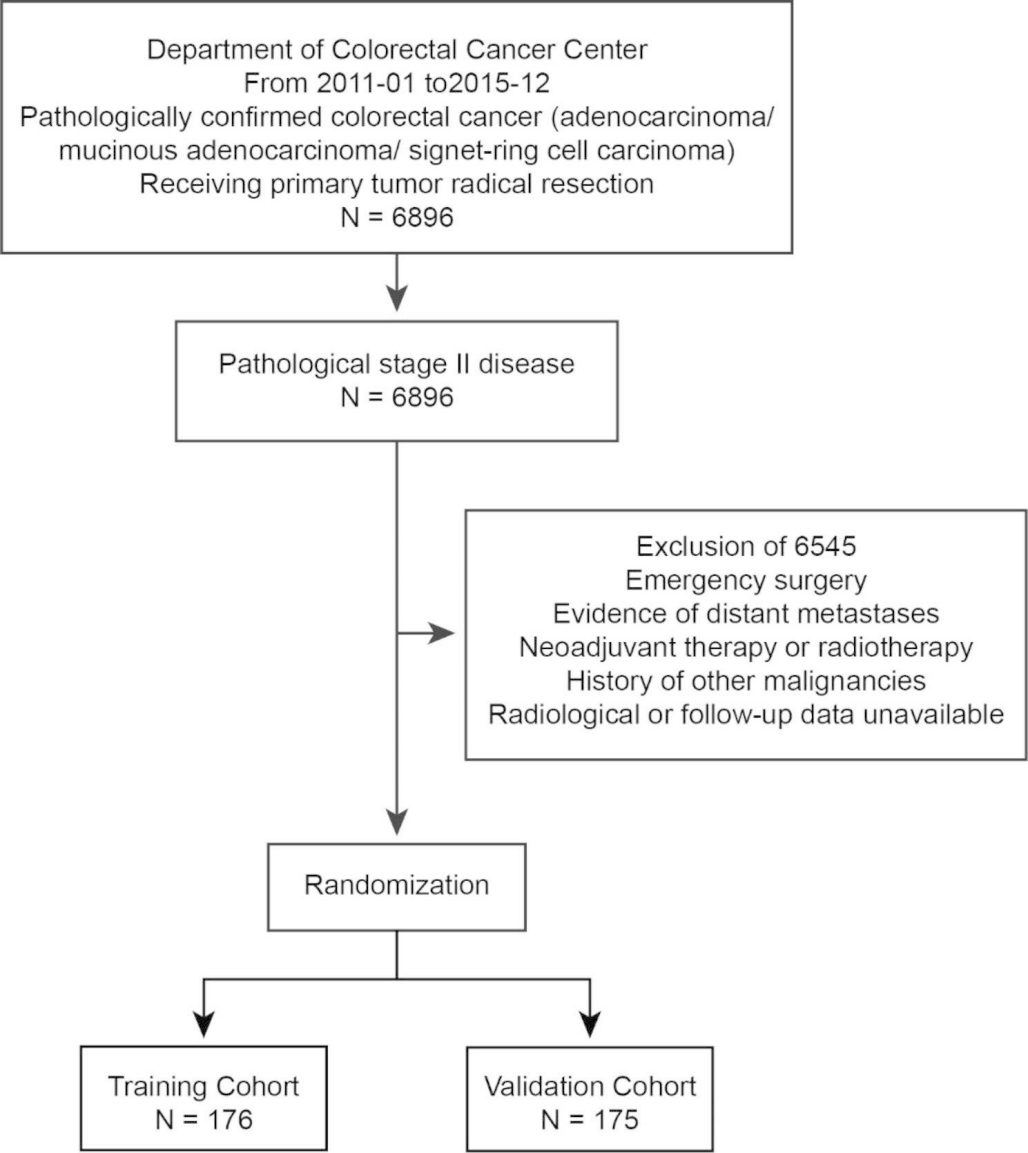
Flow diagram demonstrating the selection process, inclusion and exclusion criteria and assignment of the training cohort and the validation cohort. The pathologic stage was reevaluated according to the 8th edition of the AJCC/UICC TNM staging system

This study consecutively enrolled 351 patients at FUSCC from January 2011 to December 2015. These patients were randomly divided into two cohorts: the training cohort (n = 176) and the validation cohort (n = 175). Details of the enrollment process are presented in the flow diagram (Fig. 2).

In the training cohort, the median age of patients was 60 years old (range, 23–82, IQR, 51, 68) and the median follow-up time was 60.8 months (range, 2.7-106.1, IQR, 54.4, 71.4). There were 32(18.2%) patients who suffered from recurrence (local recurrence or distant metastases), and 15(8.5%) patients died. The 3-year RFS and OS rates were 90.3% and 94.8% respectively, and the 5-year RFS and OS rates was 81.6%, and 91.0% respectively. In the validation cohort, the median age of patients was 61 years old (range, 31–86, IQR, 53, 68) and the median follow-up time was 61.0 months (range, 1.8-107.9, IQR, 54.0, 75.3). 42(24.0%) patients suffered from recurrence, and 19(10.9%) patients died. The 3-year RFS and OS rates were 88.0% and 93.6% respectively, the 5-year RFS and OS rates were 77.5%, and 88.9% respectively. Sex, histology, tumor location, T stage, adjuvant therapy, pathological grading, venous/perineural invasion, preoperative carcinoembryonic antigen (CEA) level, and MMR (Mismatch Repair) status in the training cohort and validation cohort are shown in Table 1.

**Table 1:** Baseline demographic and clinicopathological characteristics of stage II CRC patients by SLNs and NLNs

Characteristic	Training cohort							Validation cohort						
	Cases	D		P	N		P	Cases	D		P	N		P
	N(%)	≤ 5.8	> 5.8		≤ 22	> 22		N(%)	≤ 5.8	> 5.8		≤ 22	> 22	
No. of patients	176(100.0)	79(44.9)	97(55.1)		127(72.2)	49(27.8)		175(100.0)	78(44.6)	97(55.4)		125(71.4)	50(28.6)	
Age				0.672			0.002				0.455			0.060
<60	86(48.9)	40(46.5)	46(53.5)		53(61.6)	33(38.4)		75(42.9)	31(41.3)	44(58.7)		48(64.0)	27(36.0)	
≥60	90(51.1)	39(43.3)	51(56.7)		74(82.2)	16(9.1)		100(57.1)	47(47.0)	53(53.0)		77(77.0)	23(23.0)	
Sex				0.587			0.023				0.841			0.559
Male	103(58.5)	48(46.6)	55(53.4)		81(78.6)	22(21.4)		104(59.4)	47(45.2)	57(54.8)		76(73.1)	28(26.9)	
Female	73(41.5)	31(42.5)	42(57.5)		46(63.0)	27(37.0)		71(40.6)	31(43.7)	40(56.3)		49(69.0)	22(31.0)	
Histology				0.373			0.809				0.225			0.904
Adenocarcinoma	149(84.7)	69(46.3)	80(53.7)		107(71.8)	20(28.2)		141(80.6)	66(46.8)	75(53.2)		101(71.6)	40(28.4)	
Mucinous tumors	27(15.3)	10(37.0)	17(63.0)		42(74.1)	7(25.9)		34(19.4)	12(35.3)	22(64.7)		24(70.6)	10(29.4)	
Tumor location				0.128			0.023				0.091			0.094
Left-sided	78(44.3)	40(51.3)	38(48.7)		63(80.8)	15(19.2)		84(48.0)	43(51.2)	41(48.8)		65(77.4)	19(22.6)	
Right-sided	98(55.7)	39(39.8)	59(60.2)		64(65.3)	34(34.7)		91(52.0)	35(38.5)	56(61.5)		60(65.9)	31(34.1)	
T stage				0.512			0.168				0.415			0.918
T3	118(67.0)	55(46.6)	63(53.4)		89(75.4)	29(24.6)		120(68.6)	51(42.5)	69(57.5)		86(71.7)	34(28.3)	
T4	58(33.0)	24(41.4)	34(58.6)		38(65.5)	20(34.5)		55(31.4)	27(49.1)	28(50.9)		39(70.9)	16(29.1)	
Adjuvant chemotherapy				0.507			0.135				0.695			0.082
No	48(27.3)	20(41.7)	28(58.3)		34(70.8)	14(29.2)		53(30.3)	26(49.1)	27(50.9)		44(83.0)	9(17.0)	
Yes	122(69.3)	55(45.1)	67(54.9)		87(71.3)	35(28.7)		119(68.0)	51(42.9)	68(57.1)		79(66.4)	40(33.6)	
Unknown	6(3.4)	4(66.7)	2(33.3)		6(100.0)	0(0)		3(1.7)	1(33.3)	2(66.7)		2(66.7)	1(33.3)	
Pathological grading				0.575			0.168				0.017			0.964
Well & moderate	142(80.7)	65(45.8)	77(54.2)		99(69.7)	43(30.3)		131(74.9)	63(48.1)	68(51.9)		94(71.8)	37(28.2)	
Poor & anaplastic	31(17.6)	12(38.7)	19(61.3)		25(80.6)	6(19.4)		38(21.7)	15(39.5)	23(60.5)		27(71.1)	11(28.9)	
Unknown	3(1.7)	2(66.7)	1(33.3)		3(100.0)	0(0)		6(3.4)	0(0)	6(100.0)		4(66.7)	2(33.3)	
Venous invasion				1.00			0.448				0.709			0.898
Negative	167(94.9)	75(44.9)	92(55.1)		122(73.1)	45(26.9)		160(91.4)	72(45.0)	88(55.0)		115(71.9)	45(28.1)	
Positive	9(5.1)	4(44.4)	5(55.6)		5(55.6)	4(44.4)		15(8.6)	6(40.0)	9(60.0)		10(66.7)	5(33.3)	
Perineural invasion				0.162			0.644				0.088			1.000
Negative	151(85.8)	71(47.0)	80(53.0)		108(71.5)	43(28.5)		154(88.0)	65(42.2)	89(57.8)		110(71.4)	44(28.6)	
Positive	25(14.2)	8(32.0)	17(68.0)		19(76.0)	6(24.0)		21(12.0)	13(61.9)	8(38.1)		15(71.4)	6(28.6)	
CEA (ng/ml)				0.217			0.576				0.571			0.608
≤5	93(52.8)	36(38.7)	57(61.3)		64(68.8)	29(31.2)		108(61.7)	48(44.4)	60(55.6)		75(69.4)	33(30.6)	
>5	71(40.3)	37(52.1)	34(47.9)		54(76.1)	17(23.9)		51(29.1)	21(41.2)	30(58.8)		37(72.5)	14(27.5)	
Unkown	12(6.8)	6(50.0)	6(50.0)		9(75.0)	3(25.0)		16(9.1)	9(56.2)	7(43.8		13(81.3)	3(18.8)	
MMR status				0.037			0.089				0.009			0.501
pMMR	134(76.1)	66(49.3)	68(50.7)		101(75.4)	33(24.6)		142(81.1)	70(49.3)	72(50.7)		103(72.5)	39(27.5)	
dMMR	42(23.9)	13(31.0)	29(69.0)		26(61.9)	16(38.1)		33(18.9)	8(24.2)	25(75.8)		22(66.7)	11(33.3)	

LNs, lymph nodes; SLNs, size of lymph nodes; NLNs, number of retrieved lymph nodes; CEA, Carcinoembryonic antigen; pMMR, proficient Mismatch Repair; dMMR, deficient Mismatch Repair

### Determination of SLNs and NLNs optimal cut-off values

SLNs and NLNs in the training cohort were used to determine the optimal cut-off value using X-tile program. The program calculated associations and χ^2^ values at any possible cut-off point by log-rank test for survival and selected the optimal cut-off value according to the highest χ^2^ value and the minimum P value. Then, the determined optimal cut-off values were verified in the validation cohort.

### Distribution of SLNs and NLNs

**Fig. 3:**
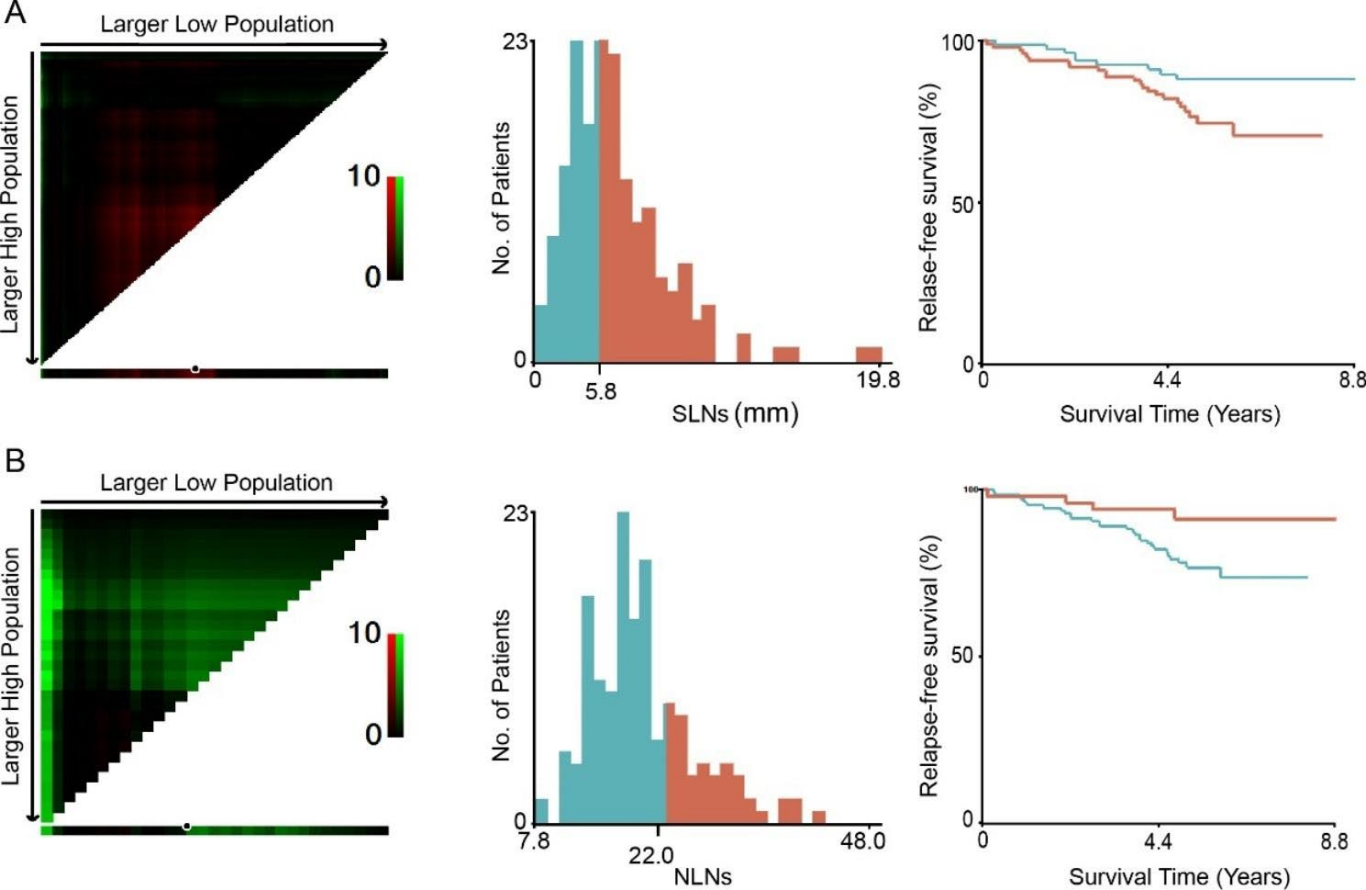
The optimal cut-off values of SLNs (**A**) and NLNs (**B**) were determined using the X-tile program in the training cohort. Red shows inverse association, while green indicates direct association for relapse-free survival

The optimal cut-off value of SLNs in the training cohort was 5.8 mm (*χ*^*2*^ = 4.4555, Fig. 3). According to the cut-off value, patients from the training cohort and the validation cohort were divided into two groups: D ≤ 5.8 mm and D > 5.8 mm. The median SLNs in the training cohort was 6.1 mm (range, 2.7–19.8, IQR, 4.9, 7.7) and in the validation cohort was 6.1 mm (range, 1.9–14.3, IQR, 4.7, 7.5). SLNs was correlated with MMR status (P = 0.037) in the training cohort. Meanwhile, it was associated with pathological grading (P = 0.017) and MMR status (P = 0.009) in the validation cohort (P = 0.001) (Table 1).

The optimal cut-off value of NLNs in the training cohort was 22 (*χ*^*2*^ = 4.5906, Fig. 3). According to the cut-off value, patients from the training cohort and the validation cohort were divided into two groups: N ≤ 22 and N > 22. The median NLNs in the training cohort was 19 (range, 7–41, IQR, 15.5, 23) and in the validation cohort was 17 (range, 0–55, IQR, 14, 23). NLNs was related to age (P = 0.002), sex (P = 0.023) and tumor location (P = 0.023) in the training cohort, whereas it was not in association with demographic or clinicopathological characteristics in the validation cohort (Table 1).

### The effect of SLNs and NLNs on RFS and OS for Stage II CRC

**Fig. 4:**
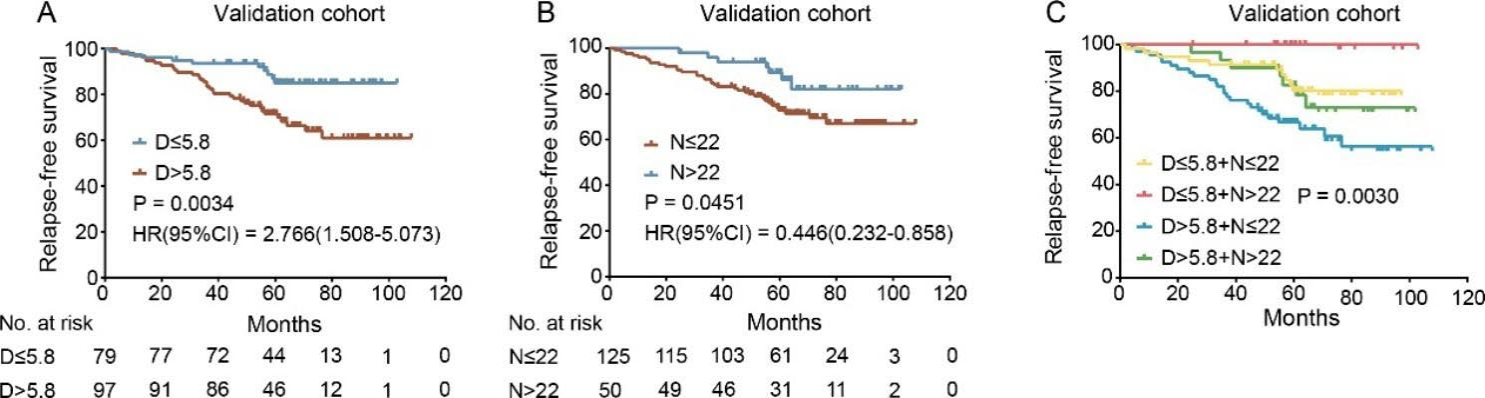
Kaplan-Meier analysis of RFS in the validation cohort. RFS according to SLNs (**A**); RFS according to NLNs (**B**); RFS according to integration of SLNs and NLNs (**C**). P values were obtained from the log-rank test and hazard ratio (HR) is calculated using GraphPad Prism

In the validation cohort, SLNs was a significant prognostic biomarker for RFS (P = 0.0034, Fig. 4A). D > 5.8 mm group were more likely to have the risk of death compared with D ≤ 5.8 mm group although this difference was not statistically significant (P = 0.4497, Supplemental Fig. 1A). Kaplan-Meier curves showed D > 5.8 mm group had a significant reduction in the 3-year RFS (93.6% vs. 83.5%), 5-year RFS (85.0% vs. 71.5%) (Fig. 4A), 3-year OS (94.8% vs. 92.6%) and 5-year OS (89.6% vs. 88.3%) (Supplemental Fig. 1A) compared with D ≤ 5.8 mm group.

NLNs was also correlated with both RFS (P = 0.0451, Fig. 4B) and OS (P = 0.0170, Supplemental Fig. 1B). Kaplan-Meier curves showed N > 22 group had a significant reduction in the 3-year RFS (96.0% vs. 84.8%), 5-year RFS (89.3% vs. 72.8%) (Fig. 4B), 3-year OS (98.0% vs. 91.8%) and 5-year OS (98.0% vs. 85.0%) (Supplemental Fig. 1B) compared with N ≤ 22 group.

Merged survival curves, divided via SLNs and NLNs, indicated that combined division by SLNs and NLNs could serve as a prognostic marker for patients as exhibited in Fig. 4C (P = 0.0030). There was similar tendency for OS (Supplemental Fig. 1C).

### SLNs and NLNs as independent prognostic factors for RFS in stage II CRC

Cox regression analysis was performed for RFS and OS in both cohorts. In the training cohort, univariate analysis showed that SLNs and NLNs (P = 0.048 and 0.043, respectively, Table 2) were associated with RFS. Multivariate analysis after adjustment revealed SLNs and NLNs (P = 0.039 and 0.049, respectively, Table 3) were also independent prognostic factors for RFS, although it was not the case for OS (Supplemental Table 2). In the validation cohort, univariate analysis showed that SLNs and NLNs (P = 0.005 and 0.049, respectively, Table 2) were correlated with RFS, and age, pathological grading and NLNs (P = 0.016, 0.032 and 0.044, respectively, Supplemental Table 1) were associated with OS. Multivariate analysis after adjustment revealed that SLNs and NLNs were identified as independent prognostic factors for RFS (P = 0.003 and 0.021, respectively, Table 3), whereas only age was an independent prognostic factor for OS (P = 0.032, Supplemental Table 2).

**Table 2:** Univariate Cox regression analysis of RFS for patients with stage II CRC in the two cohorts

Variables	Training cohort(n = 176)			Validation cohort(n = 175)		
	Hazard ratio	95%CI	P	Hazard ratio	95%CI	P
Age			0.902			0.304
< 60	1.00			1.00		
≥60	0.957	0.479–1.915		1.388	0.743–2.591	
Sex			0.905			0.874
Male	1.00			1.00		
Female	0.958	0.473–1.940		1.051	0.567–1.948	
Histology			0.616			0.230
Adenocarcinoma	1.00			1.00		
Mucinous tumors	0.765	0.268–2.182		1.524	0.766–3.032	
Tumor location			0.596			0.976
Left-sided	1.00			1.00		
Right-sided	0.829	0.415–1.658		0.991	0.541–1.816	
T stage			0.572			0.387
T3	1.00			1.00		
T4	1.228	0.603–2.502		0.742	0.377–1.459	
Adjuvant chemotherapy			0.405			0.245
No	1.00			1.00		
Yes	1.464	0.597–3.586		1.554	0.739–3.268	
Pathological grading			0.735			0.145
Well and moderate	1.00			1.00		
Poor and anaplastic	1.167	0.477–2.856		1.654	0.841–3.255	
Venous invasion			0.551			0.123
Negative	1.00			1.00		
Positive	0.546	0.074–3.999		1.978	0.832–4.706	
Perineural invasion			0.848			0.538
Negative	1.00			1.00		
Positive	0.903	0.317–2.575		0.723	0.258–2.027	
CEA (ng/ml)			0.606			0.412
≤5	1.00			1.00		
>5	0.826	0.401–1.702		0.738	0.357–1.525	
MMR status			0.598			0.380
pMMR	1.00			1.00		
dMMR	0.823	0.399–1.697		1.375	0.676–2.797	
SLNs			0.048			0.005
D ≤ 5.8	1.00			1.00		
D > 5.8	2.179	1.008–4.711		2.768	1.361–5.632	
NLNs			0.043			0.049
N ≤ 22	1.00			1.00		
N > 22	0.339	0.119–0.966		0.446	0.198–1.004	

LNs, lymph nodes; SLNs, size of lymph nodes; NLNs, number of retrieved lymph nodes; CEA, Carcinoembryonic antigen; pMMR, proficient Mismatch Repair; dMMR, deficient Mismatch Repair

**Table 3:** Multivariate Cox regression analysis of RFS for patients with stage II CRC in the two cohorts

Variables	Training cohort(n = 176)			Validation cohort(n = 175)		
	Hazard ratio	95%CI	P	Hazard ratio	95%CI	P
Age			0.747			0.339
< 60	1.00			1.00		
≥60	0.886	0.425–1.846		1.396	0.705–2.765	
Sex			0.958			0.914
Male	1.00			1.00		
Female	0.980	0.466–2.060		0.965	0.505–1.843	
Histology			0.570			0.733
Adenocarcinoma	1.00			1.00		
Mucinous tumors	0.724	0.238–2.204		1.177	0.462–2.996	
T stage			0.499			0.717
T3	1.00			1.00		
T4	1.292	0.615–2.714		0.879	0.438–1.764	
Pathological grading			0.905			0.448
Well and moderate	1.00			1.00		
Poor and anaplastic	1.060	0.410–2.741		1,397	0.589–3.313	
SLNs			0.039			0.003
D ≤ 5.8	1.00			1.00		
D > 5.8	2.361	1.044–5.338		2.979	1.435–5.184	
NLNs			0.049			0.021
N ≤ 22	1.00			1.00		
N > 22	0.335	0.113–0.994		0.375	0.156-0.900	

LNs, lymph nodes; SLNs, size of lymph nodes; NLNs, number of retrieved lymph nodes

## Discussion

To minimize the variation of SLNs, we took the largest short-axis diameter of all LNs as SLNs for each patient, which was measured by CT using a reproducible method and also applied in clinical practice [22]. The experience of radiologists and different imaging techniques such as MRI and PET-CT could impact the measurement of SLNs. The optimal cut-off points of SLN measured by MRI and PET-CT may be altered, which warrants further investigation. We evaluated the optimal cut-off value of SLNs measured by CT for RFS in stage II CRC, which was 5.8 mm. Patients whose SLNs were > 5.8 mm had poorer prognosis compared with those in whom SLNs were ≤ 5.8 mm. Bruno Märkl et al. reported that the retrieval of less than seven LNs with a long axis diameter of ≥ 5 mm was related to poorer outcomes than the retrieval of seven or more LNs of the same size in patients with stage I or II colon cancer [2, 6, 7]. Murphy et al. showed that Dukes B rectal cancer patients with the mean long axis diameter of LNs measuring < 4 mm had poorer outcomes than those in whom the mean long axis diameter of LNs were ≥ 4 mm[23]. These discordances, however, were probably due to the differences in colon or rectal cancer specificity, median follow-up time, number of enrolled cases, lymph node recovery quality and cross-validation or adjustment by multivariable analysis. Therefore, the prognostic value of SLNs for stage II CRC needs further verification.

There is incongruity in the optimal LN evaluation for stage II CRC. According to the current guideline, at least 12 lymph nodes are required to ensure accurate staging [11]. However, thresholds for optimal survival were variable, which were affected by the technique of pathology examination, the experience of surgeon and pathologist, the extent of surgical field and patient-related factors. We identified that 22 lymph nodes were the optimal threshold for RFS in stage II CRC patients, which was consistent with Xishan Wang groups’ report [24] and similar with recommendations of Hok Kwok Choi et al. [13], J. C. Del Paggio et al. and Le Voyer TE et al. [25, 26]. Patients whose NLNs were > 22 had better prognosis compared with those in whom NLNs were ≤ 22. It should be emphasized that that a proper and extensive LN search should always be performed. Additional techniques such as fat clearance and sentinel node procedures with methylene blue staining could increase LNs harvest, which can help detect very small lymph nodes that escape manual tactile detection, so the optimal NLNs may be higher for patients using these methods.

Stage migration caused by missed lymph node metastases was a prevailing theory for the association between LNs yield and survival, which was increasingly challenged by the explanation of immune response[27]. In this study, our data support the hypothesis that an immunological effect instead of stage migration is the true reason for the prognostic effect of LNs count in CRC. Firstly, most of our patients had more than 12 LNs, thus evaded the probability of stage migration to a great extent, but still a portion of them relapsed. Secondly, our data showed that both SLNs and NLNs were associated with survival. Integration of the two factors could better identify the risk of recurrence in stage II CRC patients than a single factor, which could not be simply explained by stage migration.

Therefore, to give a potential explanation of our findings, we hypothesized that patients with “large and decreased” LNs might be a surrogate marker for exhausted immune response resulting in inferior RFS compared with those with “small and increased” LNs among stage II colorectal cancers, which indicated that initial immunologic heterogeneity of CRC determined their distinct immune response pattern and pre-metastatic immune microenvironment.

Although this study has provided a new finding, we are aware that our research has some limitations. Firstly, this study is retrospective and subject to all limitations of retrospective design. Secondly, our research is a single-center cohort study. While we performed internal cohort verification to avoid overinterpretation, multiple-cohort validation is more appropriate to verify whether the findings of this study are generalizable.

## Conclusion

In conclusion, this study explored the optimal cut-off values of SLNs and NLNs for stage II CRC patients based on the prediction of RFS. We demonstrated that SLNs and NLNs were independent prognostic factors for RFS in stage II CRC patients. Though not all statistically significant, there were similar tendency for OS. Merged survival curve indicated that stage II CRC patients with “small and increased” LNs had the best RFS while those with “large and decreased” LNs were prone to have the poorest RFS, which was the same case for OS despite no significance.

## Electronic supplementary material

Below is the link to the electronic supplementary material.

Supplementary Material 1

Supplementary Material 2

Supplementary Material 3

## Data Availability

All data generated or analysed during this study are included in this published article and its supplementary information files.

## Abbreviations

LNs: lymph nodes
CRC: colorectal cancer
SLNs: size of lymph nodes
CT: computer tomography
NLNs: number of retrieved LNs
RFS: relapse-free survival
OS: overall survival
FUSCC: Fudan University Shanghai Cancer Center
HR: hazard ratio
CI: confidence interval
AJCC: American Joint Committee on Cancer
CSCO: Chinese Society of Clinical Oncology
CEA: carcinoembryonic antigen
MMR: mismatch repair
PACS: picture archiving and communication system
DICOM: digital imaging and communication in medicine
ROIs: regions of interests
