# Supplementary material for: Size and number of lymph nodes were risk factors of recurrence in stage II colorectal cancer
Source: BMC Cancer. 2023 Jun 6;23:518. doi: 10.1186/s12885-023-10935-x (PMC10243026; doi:10.1186/s12885-023-10935-x)
Supplement: Supplementary file 1 — Supplementary Material 1 [file 12885_2023_10935_MOESM1_ESM.docx]

Supplemental Table 1 Univariate Cox regression analysis of OS for patients with stage Ⅱ CRC in the two cohorts

| Variables | Training cohort(n=176) | | | Validation cohort(n=175) | | |
| --- | --- | --- | --- | --- | --- | --- |
|  | Hazard ratio | 95%CI | P | Hazard ratio | 95%CI | P |
| Age |  |  | 0.489 |  |  | 0.016 |
| <60 | 1.00 |  |  | 1.00 |  |  |
| ≥60 | 1.440 | 0.512-4.045 |  | 4.594 | 1.336-15.795 |  |
| Sex |  |  | 0.860 |  |  | 0.791 |
| Male | 1.00 |  |  | 1.00 |  |  |
| Female | 0.911 | 0.324-2.562 |  | 1.131 | 0.454-2.816 |  |
| Histology |  |  | 0.859 |  |  | 0.159 |
| Adenocarcinoma | 1.00 |  |  | 1.00 |  |  |
| Mucinous tumors | 0.874 | 0.197-3.875 |  | 2.003 | 0.761-5.272 |  |
| Tumor location |  |  | 0.538 |  |  | 0.402 |
| Left-sided | 1.00 |  |  | 1.00 |  |  |
| Right-sided | 0.727 | 0.264-2.005 |  | 0.677 | 0.272-1.684 |  |
| T stage |  |  | 0.073 |  |  | 0.200 |
| T3 | 1.00 |  |  | 1.00 |  |  |
| T4 | 2.599 | 0.915-7.383 |  | 1.811 | 0.730-4.494 |  |
| Adjuvant chemotherapy |  |  | 0.829 |  |  | 0.327 |
| No | 1.00 |  |  | 1.00 |  |  |
| Yes | 1.153 | 0.316-4.203 |  | 0.616 | 0.234-1.622 |  |
| Pathological grading |  |  | 0.821 |  |  | 0.032 |
| Well and moderate | 1.00 |  |  | 1.00 |  |  |
| Poor and anaplastic | 0.841 | 0.186-3.795 |  | 2.720 | 1.093-6.771 |  |
| Venous invasion |  |  | 0.540 |  |  | 0.218 |
| Negative | 1.00 |  |  | 1.00 |  |  |
| Positive | 0.046 | 0.0001-876.635 |  | 2.173 | 0.631-7.482 |  |
| Perineural invasion |  |  | 0.992 |  |  | 0.801 |
| Negative | 1.00 |  |  | 1.00 |  |  |
| Positive | 1.007 | 0.227-4.467 |  | 0.828 | 0.191-3.588 |  |
| CEA(ng/ml) |  |  | 0.787 |  |  | 0.359 |
| ≤5 | 1.00 |  |  | 1.00 |  |  |
| >5 | 0.867 | 0.308-2.439 |  | 0.594 | 0.196-1.807 |  |
| MMR status |  |  | 0.366 |  |  | 0.157 |
| pMMR | 1.00 |  |  | 1.00 |  |  |
| dMMR | 1.668 | 0.570-4.883 |  | 0.234 | 0.031-1.750 |  |
| SLNs |  |  | 0.325 |  |  | 0.452 |
| D≤5.8 | 1.00 |  |  | 1.00 |  |  |
| D>5.8 | 1.715 | 0.586-5.020 |  | 1.430 | 0.563-3.632 |  |
| NLNs |  |  | 0.083 |  |  | 0.044 |
| N≤22 | 1.00 |  |  | 1.00 |  |  |
| N>22 | 0.166 | 0.022-1.263 |  | 0.127 | 0.017-0.950 |  |

LNs, lymph nodes; SLNs, size of lymph nodes; NLNs, number of retrieved lymph nodes; CEA, Carcinoembryonic antigen; pMMR, proficient Mismatch Repair; dMMR, different Mismatch Repair.
