# Supplementary material for: Size and number of lymph nodes were risk factors of recurrence in stage II colorectal cancer
Source: BMC Cancer. 2023 Jun 6;23:518. doi: 10.1186/s12885-023-10935-x (PMC10243026; doi:10.1186/s12885-023-10935-x)
Supplement: Supplementary file 2 — Supplementary Material 2 [file 12885_2023_10935_MOESM2_ESM.docx]

Supplemental Table 2 Multivariate Cox regression analysis of OS for patients with stage Ⅱ CRC in both cohorts

| Variables | Training cohort(n=176) | | | Validation cohort(n=175) | | |
| --- | --- | --- | --- | --- | --- | --- |
|  | Hazard ratio | 95%CI | P | Hazard ratio | 95%CI | P |
| Age |  |  | 0.440 |  |  | 0.032 |
| <60 | 1.00 |  |  | 1.00 |  |  |
| ≥60 | 1.606 | 0.483-5.345 |  | 4.028 | 1.130-14.335 |  |
| Sex |  |  | 0.895 |  |  | 0.785 |
| Male | 1.00 |  |  | 1.00 |  |  |
| Female | 0.926 | 0.297-2.888 |  | 1.143 | 0.438-2.979 |  |
| Histology |  |  | 0.958 |  |  | 0.297 |
| Adenocarcinoma | 1.00 |  |  | 1.00 |  |  |
| Mucinous tumors | 1.045 | 0.203-5.367 |  | 1.849 | 0.582-5.874 |  |
| T stage |  |  | 0.060 |  |  | 0.139 |
| T3 | 1.00 |  |  | 1.00 |  |  |
| T4 | 2.998 | 0.954-9.424 |  | 2.032 | 0.795-5.199 |  |
| Pathological grading |  |  | 0.541 |  |  | 0.397 |
| Well and moderate | 1.00 |  |  | 1.00 |  |  |
| Poor and anaplastic | 0.602 | 0.118-3.064 |  | 1.615 | 0.532-4.898 |  |
| LNs |  |  | 0.332 |  |  | 0.249 |
| D≤5.8 | 1.00 |  |  | 1.00 |  |  |
| D>5.8 | 1.809 | 0.546-5.995 |  | 1.799 | 0.663-4.885 |  |
| NLNs |  |  | 0.113 |  |  | 0.057 |
| N≤22 | 1.00 |  |  | 1.00 |  |  |
| N>22 | 0.185 | 0.023-1.487 |  | 0.141 | 0.019-1.060 |  |

LNs, lymph nodes; SLNs, size of lymph nodes; NLNs, number of retrieved lymph nodes.
