# Supplementary material for: Size and number of lymph nodes were risk factors of recurrence in stage II colorectal cancer
Source: BMC Cancer. 2023 Jun 6;23:518. doi: 10.1186/s12885-023-10935-x (PMC10243026; doi:10.1186/s12885-023-10935-x)
Supplement: Supplementary file 3 — Supplementary Material 3 [file 12885_2023_10935_MOESM3_ESM.docx]

| 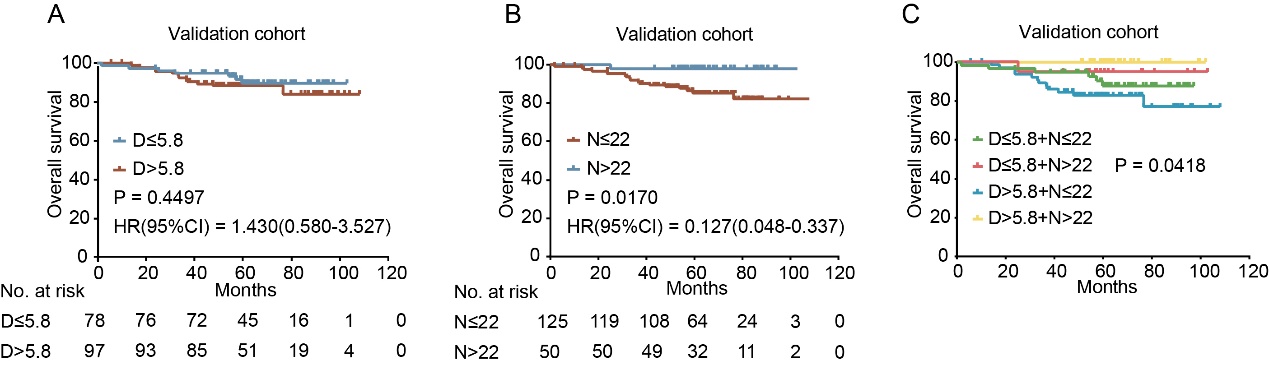 |
| --- |
| Supplemental Figure 1. Kaplan-Meier analysis of OS in the validation cohort. OS according to SLNs (A); OS according to NLNs (B); OS according to integration of SLNs and NLNs (C). P values were obtained from the log-rank test and hazard ratio (HR) is calculated using GraphPad Prism. |
